# Alpine Crossroads or Origin of Genetic Diversity? Comparative Phylogeography of Two Sympatric Microgastropod Species

**DOI:** 10.1371/journal.pone.0037089

**Published:** 2012-05-14

**Authors:** Alexander M. Weigand, Markus Pfenninger, Adrienne Jochum, Annette Klussmann-Kolb

**Affiliations:** 1 Department of Phylogeny and Systematics, Goethe University, Frankfurt am Main, Hesse, Germany; 2 Molecular Ecology Group, Biodiversity and Climate Research Centre by Senckenberg Gesellschaft für Naturforschung and Goethe University, Frankfurt am Main, Hesse, Germany; Monash University, Australia

## Abstract

The Alpine Region, constituting the Alps and the Dinaric Alps, has played a major role in the formation of current patterns of biodiversity either as a contact zone of postglacial expanding lineages or as the origin of genetic diversity. In our study, we tested these hypotheses for two widespread, sympatric microgastropod taxa – *Carychium minimum* O.F. Müller, 1774 and *Carychium tridentatum* (Risso, 1826) (Gastropoda, Eupulmonata, Carychiidae) – by using COI sequence data and species potential distribution models analyzed in a statistical phylogeographical framework. Additionally, we examined disjunct transatlantic populations of those taxa from the Azores and North America. In general, both *Carychium* taxa demonstrate a genetic structure composed of several differentiated haplotype lineages most likely resulting from allopatric diversification in isolated refugial areas during the Pleistocene glacial periods. However, the genetic structure of *Carychium minimum* is more pronounced, which can be attributed to ecological constraints relating to habitat proximity to permanent bodies of water. For most of the *Carychium* lineages, the broader Alpine Region was identified as the likely origin of genetic diversity. Several lineages are endemic to the broader Alpine Region whereas a single lineage per species underwent a postglacial expansion to (re)colonize previously unsuitable habitats, e.g. in Northern Europe. The source populations of those expanding lineages can be traced back to the Eastern and Western Alps. Consequently, we identify the Alpine Region as a significant ‘hot-spot’ for the formation of genetic diversity within European *Carychium* lineages. Passive dispersal via anthropogenic means best explains the presence of transatlantic European *Carychium* populations on the Azores and in North America. We conclude that passive (anthropogenic) transport could mislead the interpretation of observed phylogeographical patterns in general.

## Introduction

Alternations of glacial and interglacial periods as results of climatic fluctuations of the Pliocene and Pleistocene periods most likely triggered the formation of current patterns of biodiversity. Species reacted to the changing environmental conditions in diverse and individualistic ways [1], [2]. Catalysts and consequences of taxon-specific responses to changes in their geographic ranges can be investigated using techniques employed via phylogeography [3], [4]. Hypotheses can be constructed leading to a deeper understanding of the existence and position of (cryptic) refugia, routes of postglacial-expansion and suture zones of secondary contact [5]–[9]. A frequent observation is the decrease in genetic diversity from the European south to north [10], which led to the ‘southern richness’ versus ‘northern poverty’ hypothesis. This condition can be ascribed to more frequently inhabited (or permanent) southern refugia, thus possessing a higher population size and genetic diversity than more northern areas, which are only occupied during favorable conditions after bottleneck events of range expansions. On the other hand, it has been shown that northern suture zones can harbor a comparable amount of genetic diversity [11]. However, because phylogeographical patterns are highly individualistic and depend upon historical contingency as well as the characteristics specific to the taxa under study (e.g. climatic tolerance, mode of dispersal), it is important to test these hypotheses for a wide range and large number of taxa [8], [12], [13].

Terrestrial gastropods are particularly suitable for the testing of phylogeographical hypotheses [14]. Their restricted active dispersal abilities, often low effective population sizes and specific habitat requirements lead to extinction rather than to habitat tracking with historical spatial-genetic patterns remaining conserved and prominent [15]–[17]. In studies dealing with European terrestrial gastropods, the Alps, even though covered under ice, have proven to have played a major role either as a glacial refugium and/or suture zone of previously isolated lineages [18]–[22].

In this study, we focused on the taxon *Carychium* (Gastropoda, Pulmonata, Carychiidae), which comprises a group of Holarctic microgastropods (shell height 1.5–2.5 mm) inhabiting superficial subterranean habitats [23], [24]. Such (micro-) environments are defined as epigeal and consist of permanently wet, aphotic zones such as shaded, humid leaf litter and crevices [25]. In Europe, two widely distributed species are recognized – *Carychium minimum* Müller, 1774 [26] and *Carychium tridentatum* (Risso, 1826) [27]. Beyond these two common taxa, endemic species or subspecies have been described using conchological characters as designators for geographic areas within Europe [28], [29].

We performed a comparative phylogeographic approach including data for the two closely related and mostly sympatric microgastropod species *Carychium minimum* and *C. tridentatum*. Our aim was to investigate the individual responses to past climatic fluctuations and to analyze whether the broader Alpine Region (Alps+Dinaric Alps) constitutes a ‘hot-spot’ (refugium) or ‘melting-pot’ (suture zone) of genetic diversity. We tested our two competing hypotheses by the application of multiple lines of evidence using independent methods based on genetic and bioclimatic data, respectively, and by the comparative investigation of co-distributed gastropod taxa. We further benefit from the hermaphroditic nature of *Carychium* gastropods [24]. Mitochondrial mutations occurring in ‘paternal’ lineages are not immediately lost and can be potentially transferred by the same individual serving as the egg donor during any subsequent mating. Thus, it can be assumed that our study design strengthens inferences drawn from mtDNA data only. Under the ‘hot-spot’ scenario, paleoclimatic conditions for the Alpine Region at the Last Glacial Maximum (LGM) in Europe should have been suitable for *Carychium* taxa and one would expect high genetic diversity, with endemic lineages and/or haplotypes to be found in this region. Furthermore, the Alpine Region could have served as an important source for postglacial population expansions [30]. On the contrary, unsuitable paleoclimatic conditions in the Alpine Region for the time at the LGM, and a subsequent postglacial recolonization by only a few lineages and/or haplotypes from more southerly located refugia (still resulting in regions of high genetic diversity after secondary contact), would favour the Alpine Region ‘melting-pot’ hypothesis. Finally, we inferred the geographic origin of disjunct transatlantic populations of both taxa.

## Methods

### Sampling and species identification

**Figure 1:**
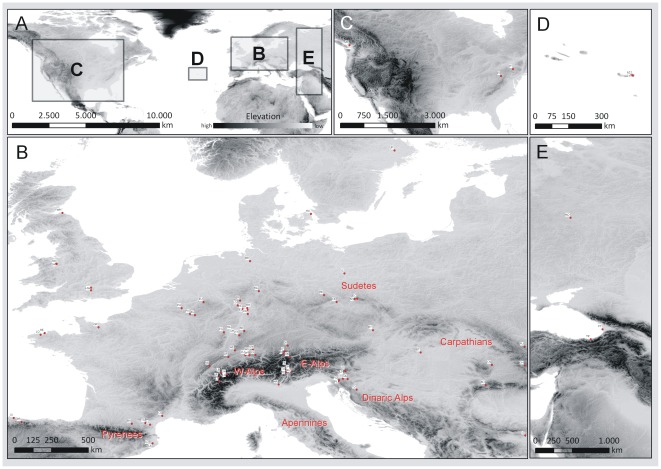
Distribution map of sampling sites. Overview map (A) of sampling sites and detailed depiction of European (B), transatlantic North American (C) and Azorean (D) as well as East European populations (E). Further information can be retrieved from Table 1.

In total, 742 specimens from 92 sampling localities (Fig. 1, Table 1) of *Carychium minimum* (CM; 325 specimens, 48 sampling localities) and *C. tridentatum* (CT; 417 specimens, 66 sampling localities) were sampled during the years 2008–2011 throughout their native European range (Fig. 1B, E) and from disjunct transatlantic sampling localities (Fig. 1C, D). No specific permits were required for the described field studies. We exclusively collected in non-protected areas and the involved *Carychium* taxa are not treated as endangered. In 24% of all populations, both species occurred in sympatry. On average, 6–7 specimens of a single species were obtained per population (minimum: 1; maximum: 15). Specimens were immediately preserved in 70–99% ethanol after collection. All individuals were identified by an integrative taxonomic approach using the combined investigation of conventional conchological characters for Carychiidae [29] and DNA barcodes [31].

**Table 1:** Locality information of analyzed sampling localities.

abb.	Locality	latitude (N)	longitude (E)	N_CM_	H_CM_	π_CM_	haplotypes CM	N_CT_	H_CT_	π_CT_	haplotypes CT
AR	France, Argentière	45.9723	6.9173					10	0.2000	0.0010	H25, H33
AS	Belgium, Assenois	49.7965	5.4557					3	0	0	H1
AU	Italy, Auer	46.3385	11.3503	9	0	0	H20	7	0.6667	0.0032	H3, H26, H29
AZ1*	Portugal, Azores	37.852	−25.265	4	0	0	H30	2	0	0	H2
AZ2*	Portugal, Azores	37.8	−25.2					8	0	0	H2
BA	Germany, Baunatal	51.2574	9.3581	7	0.7619	0.0016	H1, H8, H13	13	0.6795	0.0033	H1, H7, H8, H25, H27
BB	Germany, Bad Herrenalb	48.8247	8.4622					6	0	0	H1
BE	France, Beg En Fry	48.70	−3.72					6	0	0	H1
BH	Germany, Bad Homburg	50.227	8.635	6	0.6000	0.0102	H1, H33				
BI	Austria, Bichlbach	47.417	10.842					9	0	0	H1
BN	France, Bonnac	43.164	1.5959					12	0.1667	0.0006	H1, H16
BO	Czech Republic, Bohemia	50.7911	15.2103	9	0	0	H1	8	0	0	H25
BR	Germany, Bremen	53.09	8.82	9	0.4167	0.0008	H3, H4, H5	3	0	0	H1
BT	Hungary, Budapest	47.5115	19.2435	8	0.4286	0.0007	H1, H3	4	0	0	H1
BU	Romania, Baru	45.5119	23.2142					5	0.4000	0.0007	H22, H23
BY	Czech Republic, Brezany	48.8736	16.3308	1	n/a	n/a	H3				
CE	Czech Republic, Cereniste	50.5944	14.1089					3	0	0	H25
CN	Romania, Cluj-Napoca	46.7613	23.5869					15	0.6762	0.0041	H21, H23, H24
CS	France, Ceyras	43.6453	3.4597	10	0	0	H33				
DA	Germany, Darmstadt	49.8704	8.6783	5	0	0	H1	8	0.5714	0.0049	H1, H25
DI	Germany, Diessen	47.94	11.11	9	0.2222	0.0004	H30, H32				
EC	Czech Republic, Echemia	50.8006	15.3581					4	0.5000	0.0042	H1, H25
EP	Italy, Eppan	46.4889	11.2464	9	0	0	H9	5	0.6000	0.0021	H19, H26
ER	Germany, Erdbach	50.6813	8.2168	10	0.7111	0.0069	H1, H2, H33	5	0	0	H1
ES	Germany, Eppstein	50.1536	8.3962					10	0.3556	0.0006	H1, H11
EU	Belgium, Eupen	50.59	5.98					4	0	0	H1
FR1	Germany, Frankfurt am Main	50.1293	8.6583	4	0.8333	0.0020	H1, H7, H11	3	0.6667	0.0068	H1, H33
FR2	Germany, Frankfurt am Main	50.1244	8.6542					2	0	0	H33
FV	Belgium, Fays Les Veneurs	49.8654	5.1615	5	0.6000	0.0010	H1, H14	2	0	0	H1
GA	Slovenia, Gradicek	45.8897	14.7756	4	0.5000	0.0009	H17, H18	2	0	0	H38
GG	Slovenia, Gornji Ig	45.9189	14.4934					10	0	0	H20
GH	Romania, Gheorgheni	46.7096	25.5978	5	0	0	H1	2	0	0	H23
GI	Spain, Gijón	43.5204	−5.6164	15	0	0	H21	1	n/a	n/a	H1
GM	France, Gambsheim	48.6821	7.9032					6	0	0	H1
GN	Germany, Gaienhofen	47.68	8.99					5	0.4000	0.0007	H1, H15
GR	France, Grimbosq	49.05	−0.44					3	0	0	H1
HA	France, Haguenau	48.8806	7.7068					10	0	0	H1
HU	Denmark, Humlebaek	55.9701	12.5426	3	0	0	H1	13	0.5128	0.0009	H1, H10
HW	United Kingdom, Hartley Wintney	51.31	−0.89	10	0	0	H1				
IT1*	USA, Ithaca	42.4008	−76.5047	9	0	0	H34				
IT2*	USA, Ithaca	42.4494	−76.4758	10	0	0	H34				
KE	Germany, Kelkheim/Eppenhain	50.17	8.39	1	n/a	n/a	H33	1	n/a	n/a	H1
LA	Spain, La Creueta	41.9683	2.8572					3	0.6667	0.0023	H26, H33
LB	Switzerland, Le Bouillet	46.2781	7.0269					10	0.4667	0.0048	H1, H33
LC	Switzerland, Les Collons	46.1799	7.3889					10	0	0	H1
LD	Slovenia, Logarska Dolina	46.41	14.636					5	0.4000	0.0041	H26, H39
LF	Germany, Linderhof	47.57	10.96					4	0	0	H1
LG	Germany, Limburg	50.39	8.07	3	1.0000	0.0125	H1, H12, H33				
LM1	Switzerland, Les Masses	46.176	7.396	2	1.0000	0.0017	H23, H24	7	0.5238	0.0053	H1, H33, H34
LM2	Switzerland, Les Masses	46.23	7.01					4	0.5000	0.0025	H25, H33
LO	France, Lopreden	48.5967	−3.97					10	0.2000	0.0003	H1, H4
LP	Slovenia, Loka Pri Mengsu	46.1426	14.5533					5	0.9000	0.0068	H3, H26, H28, H30
LS	France, Les Palais	43.1080	2.7127					4	0	0	H25
LT	Italy, La Thuile	45.717	6.9445					9	0	0	H1
MA	Italy, Manerba	45.5588	10.5617	10	0	0	H33				
MO	Russia, Moscow	55.77	37.79	5	0	0	H1				
OB	Austria, Oberperfuss	47.2332	11.2443					8	0.2500	0.0030	H1, H18
OF	Switzerland, Oberdorf	47.24	7.50					5	0	0	H1
OT1	Germany, Ottenhöfen	48.5675	8.1561	8	0.6786	0.0063	H1, H31, H33, H34				
OT2	Germany, Ottenhöfen	48.5618	8.1437					6	0	0	H1
PA	Italy, Partschins	46.6901	11.0607					9	0.2222	0.0004	H3, H5
PF1	Switzerland, Pfäffikersee	47.341	8.775	3	0	0	H34				
PF2	Switzerland, Pfäffikersee	47.336	8.761	6	0.3333	0.0006	H34, H37				
PG	France, Phalsbourg	48.7506	7.2221					6	0.3333	0.0028	H1, H25
PH	Belgium, Philippeville	50.2209	4.647	2	0	0	H1	9	0	0	H1
PI*	USA, Pittsburgh	40.4367	−79.9481	3	0	0	H39				
PL	Slovenia, Planina	45.825	14.248	10	0.6444	0.0078	H27, H33				
PO	Slovenia, Postojna	45.7769	14.2036	6	0	0	H33				
PR	Spain, Precendi	43.2692	−5.1391					10	0.6444	0.0018	H26, H32, H33, H35
PT	Georgia, Poti	42.1457	41.6974	4	0.6667	0.0057	H15, H16				
PU	Romania, Putna	47.8682	25.5828	10	0	0	H25				
RE	Germany, Reinsberg	51.0180	13.3397	10	0	0	H1	4	0.5000	0.0025	H25, H33
SC	Spain, Santa Coloma de Farners	41.8581	2.6599					14	0	0	H31
SG	Switzerland, St. Gallen	47.36	9.14					8	0.1250	0.0004	H1, H14
SH	United Kingdom, Shropshire	52.9361	−3.0239					9	0.3889	0.0014	H1, H12, H13
SL	Poland, Słubice	52.3467	14.5806	10	0.7333	0.0019	H1, H3, H6, H10				
SM	United Kingdom, St. Martins	52.9244	−2.9975					8	0.2500	0.0004	H1, H3
SO	United Kingdom, Sonning	51.47	−0.89	3	0	0	H1				
SZ	Bulgaria, Stara Zagora	42.4428	25.6386	10	0	0	H1				
TE	Italy, Terres	46.3142	11.02					4	0.5000	0.0025	H17, H19
TO	Croatia, Tounj	45.2439	15.3253	6	0	0	H19	5	0.4000	0.0027	H36, H37
TR	Turkey, Trabzon	40.935	40.246	3	0.6667	0.0023	H28, H29				
TS	France, Trebes	43.2049	2.4297					7	0	0	H1
TU	Italy, Tuenno	46.3261	11.0114	10	0	0	H26				
UP1	Sweden, Uppsala	59.8532	17.6408	5	0	0	H1				
UP2	Sweden, Uppsala	59.8518	17.6288	4	0	0	H1				
VA	Switzerland, Vallorbe	46.6981	6.3456					6	0.3333	0.0034	H1, H33
VR*	Canada, Vancouver	49.24	−123.11					5	0	0	H1
WE	Germany, Wehr	47.6388	7.8972	10	0.6222	0.0106	H6, H22, H30	5	0	0	H1
WH	United Kingdom, Whitekirk	56.0433	−2.6375	10	0	0	H1	2	0	0	H9
WU	Switzerland, Wülflingen	47.52	8.70					6	0.3333	0.0006	H1, H6
ZU	Switzerland, Zürich	47.3586	8.5364	10	0.6000	0.0039	H1, H30, H38				

Provided are locality abbreviation (abb.; corresponding to Figure 1), geographic region of sampling locality (locality), georeference data (latitude, longitude), number of specimens (N), haplotype diversity (H), genetic diversity (π) and taxon-specific haplotypes (H1-39 for both taxa). CM = *Carychium minimum*; CT = *Carychium tridentatum*. Transatlantic *Carychium* sampling localities are marked by an asterisk.

### DNA extraction, amplification and sequencing

DNA of freshly conserved specimens was extracted using the DNeasy Blood and Tissue Kit (Qiagen, Hilden, Germany) and the respective protocol. Prior to DNA extraction, visceral and shell material were removed to minimize contamination risk. E-voucher data (images, georeferences, and sequence information) can be obtained from the projects ‘Phylogeography of *Carychium*’ (PHYCA) and ‘Barcoding Carychiidae microsnails’ (BARCA) stored at the Barcode of Life Data System (BOLD) [32]. The mitochondrial-encoded Folmer-fragment of the cytochrome c oxidase subunit I (COI) was amplified by polymerase chain reaction (PCR) using the standard invertebrate primer pair LCO1490 – 5′GGTCAACAAATCATAAAGATATTGG3′ and HCO2198 – 5′TAAACTTCAGGGTGACCAAAAAATCA3′ [33]. Each 25 µL PCR mixture included 1 µL (10 pmol) of each primer, 2.5 µL 10× PCR buffer, 2 µL (100 mM) MgCl_2_, 0.3 µL (20 mM) dNTPs, 0.3 µL *Taq*-polymerase, 0.25 µL (0.5 M) tetramethylammonium chloride, 1.5 µL (10 mg/mL) bovine serum albumin, 11.15 µL ddH_2_O and 5 µL template DNA. PCR cycles were run at the following conditions: 1 min at 95°C, followed by 30 cycles of 30 s at 95°C, 30 s at 52°C and 30 s at 72°C, and finally, 3 min at 72°C. Single PCR products were visualized on a 1.4% agarose gel and cleaned with the GeneJET PCR Purification Kit (Fermentas, St. Leon-Rot, Germany). In cases where multiple PCR products were detected the QIAquick Gel Extraction protocol (Qiagen) was used. PCR products were bidirectionally sequenced using the PCR primer pair and the BigDye® Terminator v.3.1 Cycle Sequencing Kit (Applied Biosystems, Inc.) on an ABI 3730 xl capillary sequencer following the manufacturer's instructions.

### Editing and alignment

Sequences were edited and aligned using Geneious 5.4 (Biomatters Ltd.) and BioEdit 7.0.9 [34] with the implemented ClustalW option [35]. In a post-editing process, primer sequences were deleted as they represent conserved (uninformative) regions influencing population genetic estimates. Further, alignment results were checked for potential 3′/5′-flanking regions of missing data by using Gblocks 0.91b [36]. The two final alignments were manually adjusted in length to receive homologous and thus comparable datasets for all two taxa.

### Population genetic analyses

Analyses of genetic data can provide insights into population structure and events shaping their demographic history, e.g. the impact of glacial periods with the formation of isolated allopatric lineages. A spatial-genetic correlation can further point to regions of high genetic diversity as potential refugial areas or zones of secondary contact.

#### Genetic and haplotype diversity

The software DnaSP v5 [37] was used to estimate the number of haplotypes per taxon as well as sampling site and to calculate the haplotype diversity (H). The genetic diversity (π) was obtained under the pairwise-deletion option in MEGA5 [38].

#### Identification of MOTUs (Molecular Operational Taxonomic Units)

Haplotype networks were constructed by using Network 4.6 (Fluxus Technology Ltd.) for Median-Joining [39] and TCS 1.21 [40] for Statistical Parsimony networks [41]. To break the mutational pathway and thus to resolve ambiguities (loops) in the networks, the approach described by [14] was followed. To identify molecular operational taxonomic units (MOTUs), the nesting design was manually constructed using the resolved haplotype networks according to the rules given in [42]. The nesting level was chosen for MOTU characterization that provided a suitable level of resolution: For example a 3^rd^ level nesting for the less genetically structured CT resulted in only two units, and would thus provide few insights into the spatial distribution of genetic diversity.

#### Demographic history

Tests for population expansion events were conducted using the software BEAST 1.6.1 [43] while comparing the CM and CT datasets under two competing scenarios – constant population size vs. exponential growth. Identical priors were used between comparable runs. Simulations were run for more than 10,000,000 generations and trees sampled every 100^th^ generation. Final samples were taken from the stationary phase of the runs. The convergence of relevant parameters was assessed using Tracer v1.5 [44]. The traces of the MCMC samples and the effective sampling size with parameters for each run showing values >100, thus indicating a sufficient level of sampling were inspected. For model comparison and selection of the best fitting model, the harmonic means of tree likelihoods were compared using a Bayes Factor (BF) test [45]. After [46], the results of log BF can be interpreted as substantial (½-1), strong (1–2) and decisive evidence (>2) for a given hypothesis.

The McDonald-Kreitman test for a combined dataset of CM+CT was conducted in DnaSP v5. This test is based on the comparison of synonymous and non-synonymous differences within and between species [47] and calculates the amount of polymorphic synonymous (Ps), polymorphic non-synonymous (Pn), fixed synonymous (Ms) and fixed non-synonymous (Mn) differences. Thereby, a site is called polymorphic if it shows variation within species, while it is classified as fixed if it differs between species but not within them. In case genetic changes are the result of positive selection, the ratio of fixed differences to polymorphisms is much higher for non-synonymous changes (Mn/Pn>>Ms/Ps).

For selected MOTUs additional Tajima's D [48] and Fu's F_S_ [49] measures for neutral molecular evolution were determined in DnaSP v5 [37]. Both statistics identify deviations from the null-hypothesis (selective neutrality) of the neutral theory model at equilibrium between genetic drift and mutation. Significant negative deviations from 0 can indicate past population expansion and/or purifying selection, whereas significant positive values can result from balancing selection and/or a decrease in population size. Tajima's D and Fu's F_S_ measures are significant below the 0.05 and 0.02 p-level, respectively, which were calculated in DnaSP v5 (1000 coalescent simulations). Deviations of the observed data from a simulated model of population expansion [50] were further tested by calculating mismatch distributions (frequency of pairwise genetic differences against stepwise genetic distance of haplotypes) and estimating the Harpending's raggedness index (Hri; [51] in DnaSP v5. A significantly high value of Hri (p<0.05) means a non-fitting deviation, and thus, a rejection of the null-hypothesis of a population expansion model.

### Species potential distribution modeling

The comparison of species potential distribution models under recent and past bioclimatic conditions can help to identify (re-)colonization events and to locate permanently suitable regions (stable habitat) that with high probability, could have served as refugial area.

Recent and past species potential distribution models were estimated using presence-only maximum entropy modeling implemented in Maxent 3.3.3 [52], [53]. This approach combines actual georeference data (presence-only points) and layers of environmental variables to create a probability distribution (potential distribution) given the parameters (taxa+bioclimatic variables) and area under study. Model runs were performed constituting 39 and 58 molecularly confirmed presence points of CM and CT, respectively. We used the 19 bioclimatic variables from the WorldClim database [54] to estimate models of ‘present-Europe’ (mean values 1950–2000) and MIROC3.2 palaeodata from [55] to generate models for the European Last Glacial Maximum (‘LGM-Europe’). Both datasets were retrieved from [56] and used in a comparable spatial resolution of 2.5 arc min. In an *a priori* selection, we screened for important environmental variables to yield an even better goodness-of-fit than a mere model containing all variables. Consequently, the spatial and temporal transferability will be enhanced if such variables are omitted from the model runs [57], [58]. The variables bio8+bio11 for CM and bio18 for CT showed significant overfitting/covariation and where excluded from the final model runs. For all models, we used 25% of presence points for model testing and performed 10 bootstrap replicates under the recommended settings (convergence threshold: 0.00001; maximum number of iterations: 500; background points: 10,000; see [57]). Presence thresholds were implemented to render continuous logistic model outputs into binary formats using ArcGIS 10.0 (ESRI® Inc.). The minimum prediction that corresponds to a given presence-point (CM: 0.1011; CT: 0.1157) was used for ‘present-Europe’ models and a 50% threshold of the maximum of the logistic entropy function (as a proxy for species potential distribution) was set for ‘LGM-Europe’ models. All models were evaluated using the area under the receiver operating characteristic curve (AUC) statistic, with values for model performance ranging from 0.5 (random) to 1 (perfect) [59], [60].

### Refugium localization reconstruction

In distinguishing between ‘hot-spot’ (refugia) and ‘melting-pot’ (secondary contact) regions, it is important to detect past population expansion events and to localize their potential origins in congruence with spatial-genetic patterns and bioclimatic data.

The refugium localization reconstruction approach (RLR) combines methods used in phylogenetic ancestral character reconstruction and population genetics for inferring the ancestral locality of the most recent common ancestor (MRCA) of a given lineage [61]. It follows the rationale that the coalescent structure of the haplotypes contains information on the origin of their MRCA if a suitable dispersal model is applied. In this case, we applied a parsimonious dispersal model. This model assumes that a) colonization events are rare; b) colonization proceeds not strictly in a stepping-stone fashion, but rather under an island model and c) secondary gene-flow among populations can be neglected. All these assumptions are probably met by land snail populations [61], [62]. By treating the sampling site as a discrete character, the dispersal routes can then be traced back in time along the coalescence tree, which has an inherent temporal direction. However, since the true coalescence tree of the sampled haplotypes is not known, a Bayesian approach was chosen to account for this uncertainty.

We used 250 randomly-sampled COI coalescent trees of the stationary phase of a BEAST-run applying the same parameters as mentioned for the demographic history analyses. BEAST-runs under constant size and exponential growth scenarios were compared using Bayes Factor tests (Table S1). Runs under constant size scenarios performed slightly better and thus were consistently used for tree sampling. For the estimation of the ancestral locality, each locality was treated as a character and assigned to the respective haplotype. To increase the resolution, adjacent localities were pooled (Table S2). Calculations for the parsimonious ancestral character reconstruction were conducted in Mesquite 2.75 [63]. Ancestral characters (n) of the MRCA were identified and, if necessary, weighted according to the total number of equivalent ancestral characters at the root in a given genealogy (w = 1/n). Finally, the relative probability score of a given locality was determined by summing up all weighted occurrences (w) divided by the total number of trees (here 250).

## Results

### Genetic and haplotype diversity

**Figure 2:**
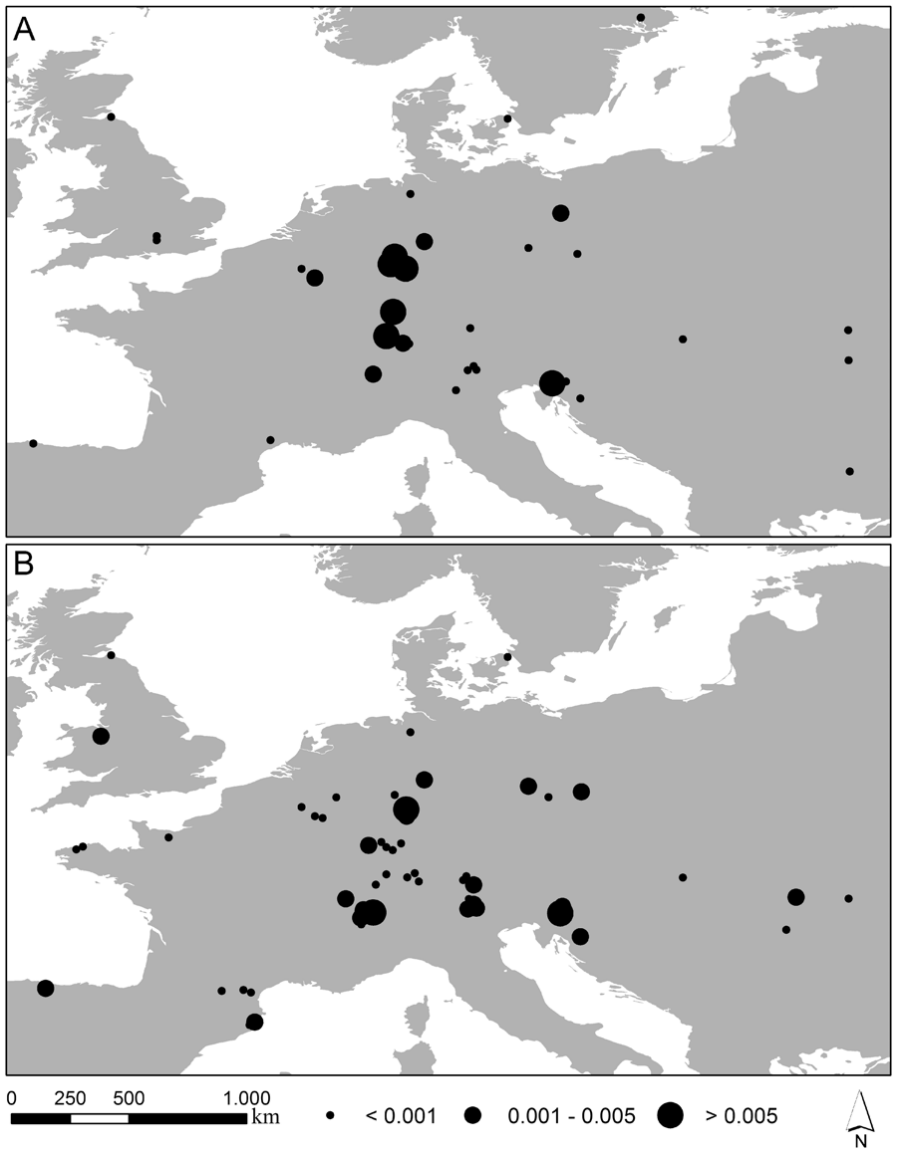
Distribution of genetic diversity of *Carychium minimum* (A) and *C. tridentatum* (B) in Europe. Genetic diversity is classified into three sections (with π<0.001; 0.001–0.005; >0.005) and indicated by the respective dot.

The COI datasets have a length of 590 homologous base pairs (bp). Total numbers of variable sites are 52 (*Carychium minimum*, CM) and 39 (*Carychium tridentatum*, CT). We detected the same number of haplotypes (n = 39) for both taxa (Table 1). All differences between haplotypes result from single nucleotide polymorphisms. Mean genetic (π) and haplotype diversity (H) are both significantly higher in CM (π_CM_ = 0.0105, SD 0.0003; H_CM_ = 0.8351, SD 0.0150) than in CT (π_CT_ = 0.0045, SD 0.0002; H_CT_ = 0.6758, SD 0.0250). The number of haplotypes per locality extends from 1–5 (Table 1). Genetic diversity per locality in CM has maximal values in western/southwestern Germany (localities BH, ER, LG and WE) and in the Dinaric Alps (PL) (Fig. 2). Sampling localities with the highest genetic diversity for CT are located in western Germany (DA, FR1), the Dinaric Alps (LP) and the Alps (LB, LM1). The four (AZ1, IT1, IT2 and PI) and three (AZ1, AZ2 and VR) transatlantic sampling localities of CM and CT (Fig. 1C, D) are represented by a single haplotype each and thus, possess a haplotype and genetic diversity of zero.

### Phylogenetic relationships and spatial analyses of haplotypes and molecular operational taxonomic units in Europe

#### *Carychium minimum*

**Figure 3:**
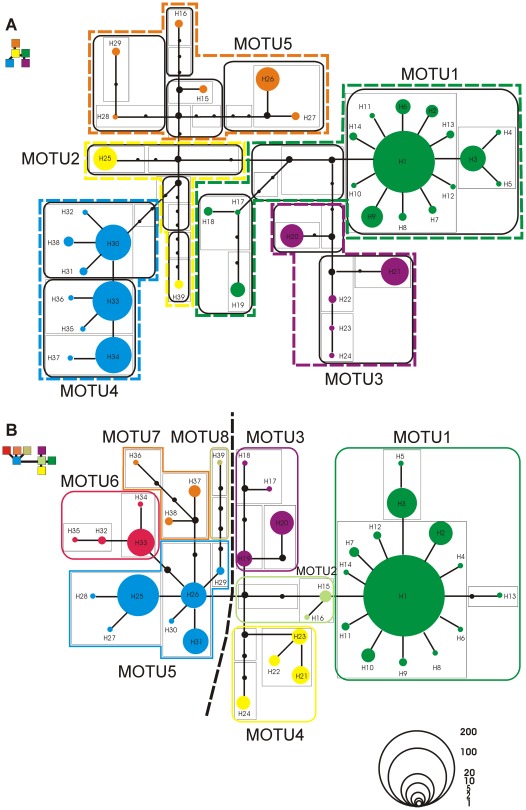
COI haplotype networks for *Carychium minimum* (A) and *C. tridentatum* (B). The respective nesting is indicated as a thin (1^st^ level), thick (2^nd^ level) and thick dotted line (3^rd^ level). The applied nesting design is color coded, with colored boxes indicating taxon-specific molecular operational taxonomic units (MOTUs). Haplotypes are numbered consecutively and possess an area relative to their frequency in the total dataset. Lines and filled circles interconnecting haplotypes represent the mutational pathway and the amount of mutational steps between them.

**Figure 4:**
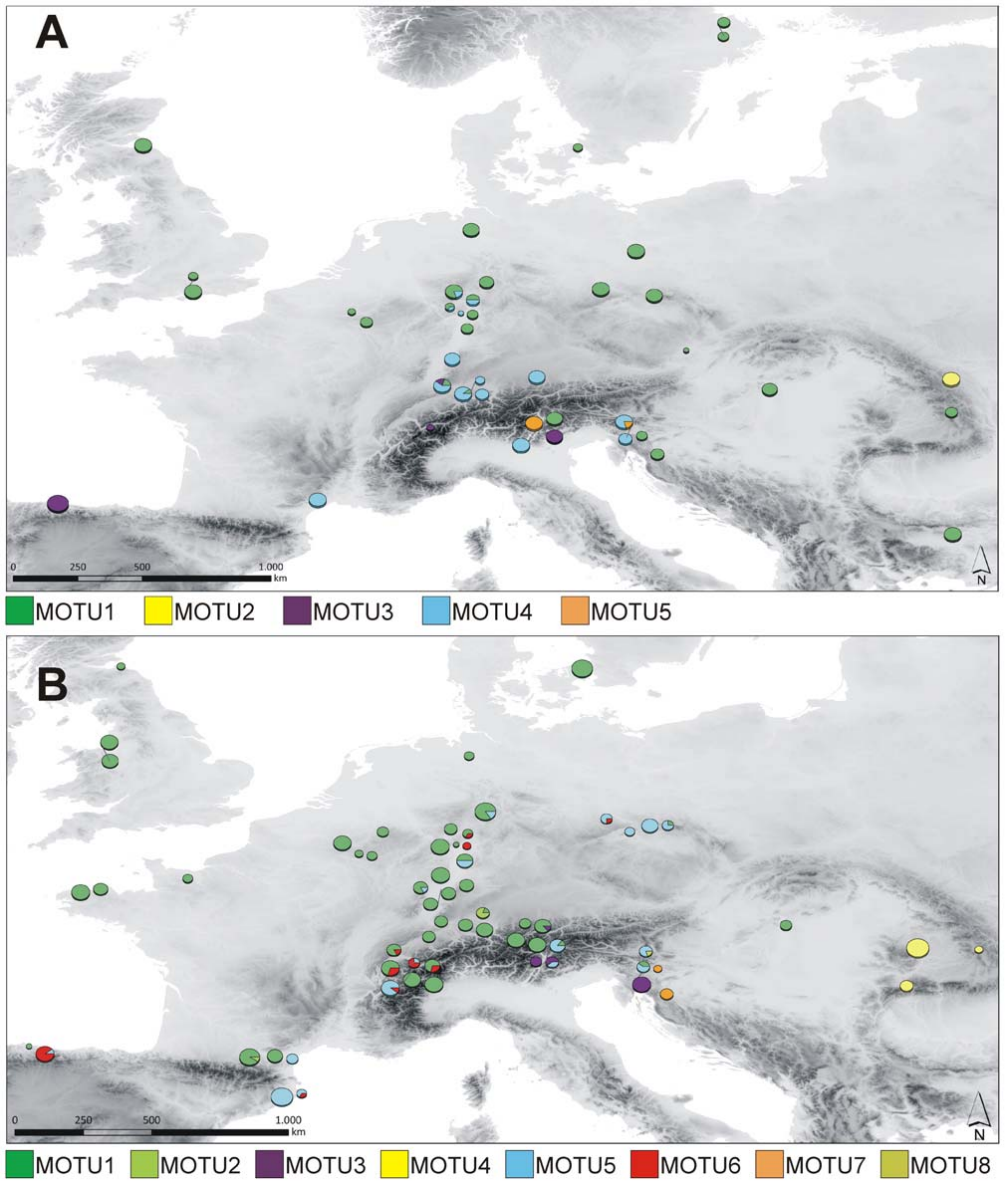
Spatial distribution of molecular operational taxonomic units (MOTUs) of *Carychium minimum* (A) and *C. tridentatum* (B) in Europe. Size of localities is proportional to analyzed specimens per population.

Results of the resolved Median-Joining and Statistical Parsimony network are identical (Fig. 3A). The observed 39 haplotypes for CM can be divided into 5 MOTUs (CM_MOTU1_ – CM_MOTU5_) following the results of a 3^rd^ level nesting. CM_MOTU1_ demonstrates a star-like topology comprising the most frequent haplotype H1 (Table 1) and is the most spatially widespread (Fig. 4A). Haplotypes are distributed over southeastern (Dinaric Alps, Balkan Mountains), Central (e.g. Germany, Belgium) and Northern Europe (e.g. Great Britain and Sweden), where in the latter region this MOTU can exclusively be found. All other MOTUs form more spatially restricted genetic entities. Among these, CM_MOTU4_ shows the widest distribution with haplotypes located in southern France, the Alps, the Dinaric Alps and south/southwestern Germany. Haplotypes of the other three MOTUs are restricted to the Carpathians (CM_MOTU2_), northern Spain (CM_MOTU3_) and the Alpine Region (CM_MOTU3_+CM_MOTU5_). Haplotypes of CM_MOTU5_ are also present at two localities on the Black Sea coast (Table 1: PT, TR).

#### *Carychium tridentatum*

Resolved Median-Joining and Statistical Parsimony networks provide the same results (Fig. 3B). As a consequence of the more shallow genetic structure (CT shows the lowest H and π), MOTUs have been assigned according to a 2^nd^ level nesting. The 39 haplotypes form 8 divergent haplotype lineages (CT_MOTU1_ – CT_MOTU8_). The application of a 3^rd^ level nesting separates CT_MOTU1-4_ from CT_MOTU5-8_ (Fig. 3b, dotted line) and would result in a very coarse spatial-genetic pattern. A geographical trend for this clustering is roughly visible along an east to west corridor, with haplotypes of CT_MOTU1-4_ and CT_MOTU5-8_ occurring more likely northwards and southwards (respectively) of this virtual barrier (Fig. 4B). Main exceptions are localities in central Europe (e.g. localities BO, DA, RE), along the French Pyrenees (BN, TS) and in northern Spain (GI). The most widespread MOTU of this taxon, CT_MOTU1_, has its main distribution in the Alps, in Central and Western Europe as well as in regions as far north as Great Britain and Denmark. In general, MOTUs are geographically restricted, with e.g. CT_MOTU3_ and CT_MOTU7_ present only in the Alpine Region and CT_MOTU4_ limited to the Carpathians. On the contrary, some MOTUs possess a geographical pattern indicating a wider but patchy distribution (e.g. CT_MOTU5_ and CT_MOTU6_).

### Phylogeography of transatlantic European *Carychium*

The seven transatlantic localities of CM (4 localities) and CT (3 localities) are characterized by only one haplotype per locality (Fig. 1C, D; marked by an asterisk in Table 1). The sampling localities of CM from the Azores (AZ1) and from Ithaca, USA (IT1, IT2) reveal haplotypes H30 and H34 which cluster within CM_MOTU4_ (Fig. 3A). Haplotype H39 of CM is endemic to Pittsburgh, USA (PI) and together with H25 from Romania constitutes the European CM_MOTU2_. In the case of CT, all individuals from the three transatlantic sampling localities belong to the same haplotype lineage, CT_MOTU1_, with specimens from the Azores (AZ1, AZ2) demonstrating haplotype H2 and individuals from Vancouver, Canada haplotype H1. Thus, six out of seven transatlantic sampling localities possess identical (CM: H30, H34; CT: H1) or closely related haplotypes (CT: H2) as found on the European mainland.

### Species potential distribution modeling

Model runs for CM (39 presence points, omitting variables bio8+bio11) and CT (58 presence points, omitting variable bio18) ‘present-Europe’ and ‘LGM-Europe’ potential distribution models performed well. The average values for training AUC of both datasets are close to 1 (CM: 0.954, SD 0.007; CT: 0.966, SD 0.006). Thus, these models possess a high predictive performance and transferability.

**Figure 5:**
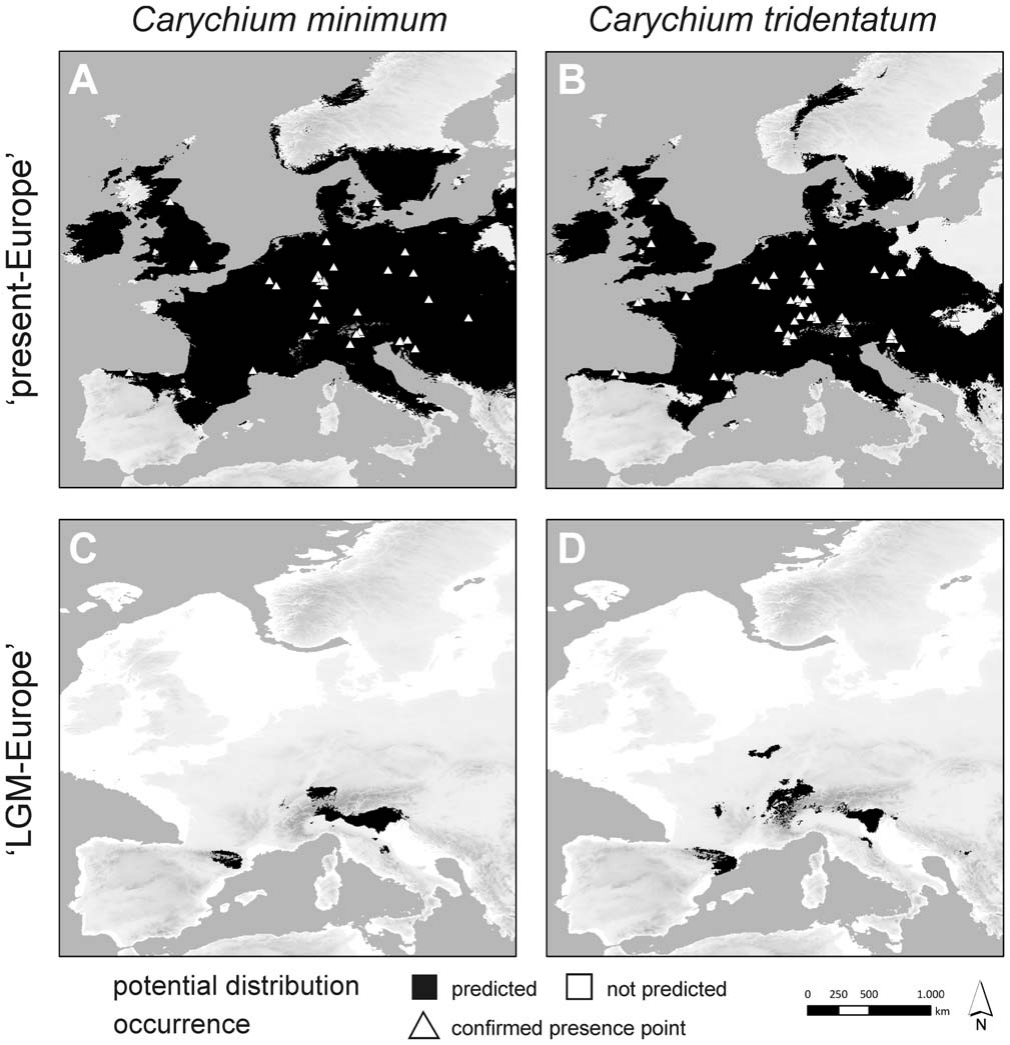
Potential distribution models of *Carychium minimum* and *C. tridentatum*. Shown are reconstructions for the bioclimatic conditions in present Europe (A, B) and during the European Last Glacial Maximum (LGM) approximately 21,000 years before present (C, D). Potential distribution is marked in black; triangles indicate the location of molecularly confirmed presence points obtained from this study.

The ‘present-Europe’ models for CM and CT demonstrate a wide area of potential distribution for each taxon (Fig. 5). Except for parts of the southern Mediterranean Peninsulas (Iberian Peninsula, Italian Peninsula, Balkan area) and regions of Scandinavia, the European mainland exhibits suitable bioclimatic conditions. Moreover, model outputs for both taxa imply a mainly sympatric distribution. Our own sampling results yielded a sympatric occurrence of CM and CT at 24% of all localities. Beyond this, model runs suggest CM occurring more to the North and East of Europe (e.g. Baltic states), whereas CT can inhabit regions slightly more to the West (e.g. Brittany, Galicia).

Potential distribution in the ‘LGM-Europe’ models is restricted to several isolated regions (Fig. 5C, D). For CM, the borders of the Alps, Apennines and Pyrenees provided suitable conditions. The same suitable areas can be recognized for CT. However, the potential distribution map for this taxon displays additional potential refugial areas. Suitable conditions have been modeled for the mountainous region of Wallonia, the upland area of the French Limousin region, and within the Dinaric Alps.

At this point, it should be highlighted that the potential distribution does not necessarily reflect the realized distribution. Hence, regions with positive model results should be regarded only as potential distribution areas including (micro-) habitats in which the taxa are able to survive.

### Demographic History

**Figure 6:**
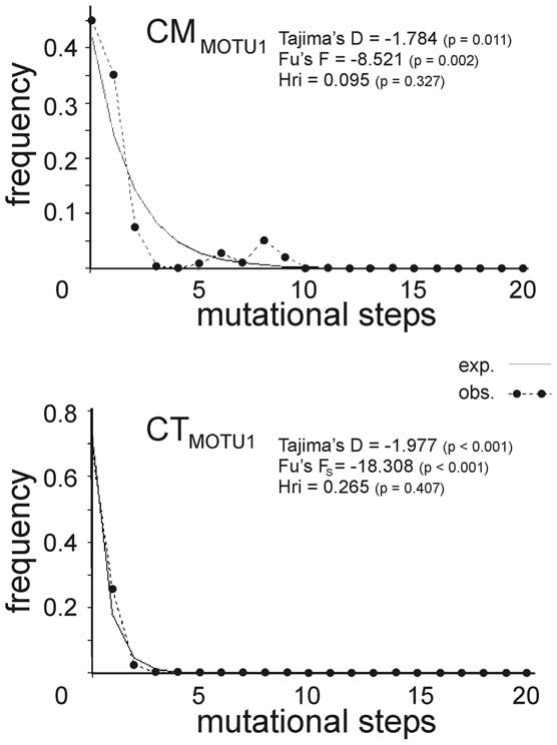
Tests of population expansion for the most widespread MOTU per taxon. Mismatch distributions and values for the neutrality tests of Fu's F_S_ and Tajima's D are provided for the haplotype lineages CT_MOTU1_ and CM_MOTU1_ (*** = p-value with significance level <0.00001). Solid and dotted lines indicate expected distributions after events of range expansion and the observed functions, respectively.

Bayes factor (BF) tests of both datasets favor a scenario of constant population size over a scenario with exponential growth (BF_CM_ = 2.099; BF_CT_ = 6.482). The complete COI-dataset of CM provides no indication for an event of past population expansion (Table 2). However, both neutrality tests for the complete dataset of CT and MOTU1 of both species are significantly negative. Haplotype lineages of CM_MOTU4_ and CT_MOTU6_ demonstrate significantly negative results for Tajima's D only. We found no MOTU to be significantly negative for Fu's F_S_ only. As shown by the respective unimodal mismatch distributions, the observed data for CM_MOTU1_ and CT_MOTU1_ are not significantly different from modeled distributions of population expansion (Fig. 6). Goodness-of-fit estimates are Hri = 0.095 (p = 0.327) and Hri = 0.265 (p = 0.407) for CM_MOTU1_ and CT_MOTU1_, respectively. The McDonald-Kreitman test revealed 71 polymorphic synonymous (Ps), 4 polymorphic non-synonymous (Pn), 6 fixed synonymous (Ms) and no fixed non-synonymous differences (Mn). Due to the lack of Mn, the McDonald-Kreitman test does not indicate that differences have been subject to positive selection. However, the selective pressure could have operated upon linked genes within the (single-molecule) mitochondrial genome. Moreover, even a significant result for positive selection does not necessarily exclude population expansion, as these differences could have enabled population expansion in the first place.

**Table 2:** Tests for neutral molecular evolution.

taxon	unit	N	H	Tajima's D	Fu's F_s_
CM	complete	325	39	−0.975	−6.461
	MOTU1	160	17	−1.784*	−8.521*
	MOTU2	13	2	0.782	7.801
	MOTU3	28	5	0.644	1.034
	MOTU4	105	9	−1.656*	−3.232
	MOTU5	19	6	0.516	2.683
CT	complete	417	39	−1.587*	−18.265*
	MOTU1	270	14	−1.977*	−18.308*
	MOTU2	5	2	−0.817	0.090
	MOTU3	18	4	−0.110	1.143
	MOTU4	22	4	1.115	2.312
	MOTU5	70	7	−0.216	−0.889
	MOTU6	24	4	−1.279*	−1.936
	MOTU7	7	3	−0.330	1.222
	MOTU8	1	1	n. c.	n. c.

Tajima's D and Fu's F_s_ neutrality tests for the complete COI-datasets and single molecular operational taxonomic units (MOTUs) of *Carychium minimum* (CM) and *C. tridentatum* (CT). Significantly deviating values from zero are indicated in bold and marked by an asterisk; n. c. = not calculable.

#### Identification of source populations of expanding lineages

**Figure 7:**
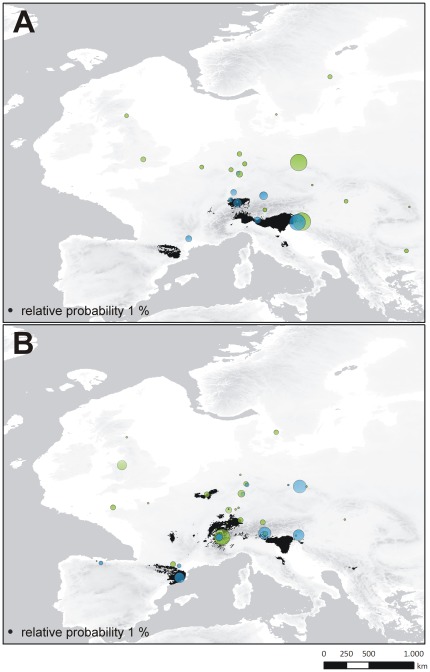
Refugia localization reconstruction. Reconstructions for the ancestral region of potentially postglacial expanding (i.e. most widespread) lineages of *Carychium minimum* (A; CM_MOTU1_ in green; CM_MOTU4_ in blue) and *C. tridentatum* (B; CT_MOTU1_ in green; CT_MOTU5_ in blue). The size of circles is proportional to the relative probability score of regional ancestry.

The inference of the ancestral locality was conducted for a) the most widely distributed and potentially expanding haplotype lineages of CM (Fig. 4A; CM_MOTU1_) and CT (Fig. 4B; CT_MOTU1_ and b) the second most widespread lineage of each taxon providing sufficient occurrence data (CM_MOTU4_, CT_MOTU5_). Results of the RLR approach reveal significant relative probability scores (RPS) for three lineages (Fig. 7). For the CM_MOTU1_ lineage, a pooled locality in the Dinaric Alps (RPS_GA+TO_: 38.7) and a pooled locality in the region of the Sudetes (RPS_BO+RE+SL_: 35.9) possess maximal scores. The pooled sample of PL+PO in the Dinaric Alps demonstrates the highest RPS (34.0) for the ancestral locality of CM_MOTU4_. A West-Alpine pooled locality (LB+LC+LM+LT) demonstrates the maximal RPS (34.3) for CT_MOTU1_. In the case of CT_MOTU5_, no clear signal is revealed with four localities showing comparable relative probability scores (RPS_BO+CE_: 25.7; RPS_AU+EP_: 23.5; RPS_LP_: 19.7 and RPS_LA+SC_: 18.4).

## Discussion

### Independent of individual responses: Alpine origin of genetic diversity

The generalness of hypotheses dealing with the location of glacial refugia, migration routes and the origin of suture zones is challenged by increased recognition of the individuality of species responses to past climatic changes [8]. Therefore, more recently performed phylogeographical studies on ‘refugia within refugia’ or ‘cryptic northern refugia’ provide even more evidence that individualistic patterns are far more diverse as initially expected [5], [6], [64]–[66]. Since gastropods are highly suitable for the study of phylogeographical patterns [14], we investigated the spatial-genetic structure of two closely related mostly sympatric European microgastropod species of the taxon *Carychium*. Our objective was to infer the presence of glacial refugia, events of postglacial recolonization and whether the Alpine Region (Alps+Dinaric Alps) constitutes an origin (‘hot-spot’) or crossroads (‘melting-pot’) of genetic diversity [11]. Individual responses as well as shared trends have been revealed by our study.

In general, genetically differentiated molecular operational taxonomic units (MOTUs) with limited geographical distribution can be identified for both *Carychium* taxa (Figs. 3, 4). We expect these MOTUs to be results of allopatric populations inhabiting geographically isolated areas during the Pleistocene glaciations. Several of these geographically restricted haplotype lineages can be exclusively found or possess haplotypes specific to the Alps or to the peri-Alpine region and the Dinaric Alps hence, providing evidence for the Alpine Region ‘hot-spot’ scenario. Examples for endemic haplotypes are provided within CM_MOTU3_ (Alps), CM_MOTU5_ (Southern Alps and Dinaric Alps), CT_MOTU3_ (Central+Eastern Alps and Dinaric Alps), CT_MOTU7_ (Dinaric Alps) and CT_MOTU8_ (Eastern Alps and Dinaric Alps). Many of these areas were likewise modeled as potential distribution for both taxa at the time of the Last Glacial Maximum (LGM) (Fig. 4C, D). This was expected under the Alpine Region ‘hot-spot’ hypothesis and thus, provides independent support for this scenario. Moreover, several postglacial expanding lineages provide evidence of ancestry within the Alps (Fig. 7), again favoring the Alpine Region ‘hot-spot’ hypothesis. In particular, the habitat heterogeneity of the Alps and Dinaric Alps could have significantly increased the possibility of persistence for several isolated lineages during glacial periods [30], [67]–[69]. Microhabitats of only a few square meters in size would have been required for long term survival of these snail species [70]. During our collecting activity in December 2008, we found active populations of CM and CT under layers of snow and ice at two localities (Fig. 1B: BA, ER). Cold-tolerant species are expected to demonstrate an even greater ability to resist the extreme climatic conditions during the LGM [13]. Finally, as several *Carychium* populations are known from North American caves [71]–[73] and specimens of CT were found in close proximity to the entrances of caves (Table 1: LP, TO), a retreat during glaciations into subterranean habitats could well have promoted glacial survival. Hence, and in congruence with former phylogeographical studies [7], [19], [67]–[69], [74], [75], we expect the Alpine Region to exhibit potential LGM refugia for *Carychium* lineages. Although the Carpathians are not modeled as a potential LGM refugium (Fig. 5C, D), the presence of specific MOTUs restricted to this area (Fig. 4: CM_MOTU2_ and CT_MOTU4_) and the independent identification of this region in former phylogeographical studies dealing with other species (e.g. [66], [75]–[77]) allows the assumption of an additional potential refugial area for *Carychium*. On the other hand, we were not able to assign MOTUs to other potentially suitable regions, such as the Pyrenees and the regions of Wallonia and Limousin (Fig. 5).

Despite overlapping potential distribution models for both taxa and time periods (Fig. 5), the genetic structure in CM is more prominent than is the case in CT (Fig. 3). A possible reason could originate from discrepancies in ecological tolerances these two taxa possess. CM is known to be a highly hygroscopic species preferably inhabiting marshes, swamps and riparian zones of permanent water systems. On the other hand, CT demonstrates a greater petrophilic affinity, is extremely tolerant of morphologically rich and diverse mountainous terrain, and is in general, less ecologically restricted. In addition to these habitats, CT can be found in ephemeral populations inhabiting moderately moist leaf litter and sink holes [23], [78]–[80]. These ecological differences in habitat preference and geomorphological demands could on the one hand, have led to enhanced isolation of CM populations and on the other, created a more continuous distribution area facilitating ongoing gene-flow between CT populations during glacial periods. Thus, accompanied by limited gene-flow in isolated habitats, genetic drift would have had a greater effect on small population sizes and result in a more pronounced genetic differentiation as is revealed for CM [81]. In addition, the potential distribution models for CM and CT (Figs. 4A, B) demonstrate a significant overlap in their distribution, whereby sampling occurred sympatrically at only 24% of all localities. This discrepancy can be explained by one or more of: microhabitat differentiation as discussed above, coarse resolution of the bioclimatic variables serving as input for the potential distribution models, and the realized distributions of the taxa within their potential distributions.

Another significant observation is the postglacial recolonization of Northern Europe performed by a single MOTU per species with generally declining genetic diversity (Figs. 2, 4, 6). The MOTU1 of CM and CT for example, present interesting scenarios. In the case of CM, our findings suggest that the postglacial expansion of MOTU1 probably originated in the Eastern Alps (Fig. 7A). The observed pronounced genetic structure of sampling localities located in the Sudetes and a high relative probability score as an ancestral region for the latter, can be either ascribed to a comprehensive postglacial (vector-mediated) colonization of this area or to the existence of cryptic microrefugia. Since our sampling within e.g. the region of the Danube valley between the Alps and the Sudetes is limited and will be subject to further investigation, both hypotheses will be discussed regarding new sampling data in subsequent work. However, our results further imply a southeast expansion of this MOTU with colonization of the Balkan area. The most widespread MOTU of CT, CT_MOTU1_, most likely stretched from the Western Alps, and was able to expand and occupy previously uninhabitable areas in Northern, Western and Southwestern Europe (Figs. 5D, 7B). Further statistically non-significant range expansions are likely for two more lineages (Fig. 4). The actual peri-Alpine distribution of CM_MOTU4_ can most likely be explained by a scenario with origin in the Eastern Alps and postglacial expansion into the broader Alpine Region (Figs. 4A, 7A). The actual distribution of lineage CT_MOTU5_ is highly irregular. We identified specimens assigned to this haplotype lineage in far distant mountain ranges such as the Pyrenees, Alps and the Sudetes (Fig. 4B). Since CT generally possesses a weak genetic structuring (Fig. 3B) and CT_MOTU5_ demonstrates a patchy distribution, the ancestral area for this lineage could not be resolved (Fig. 7B).

Finally, we detected several regions and even sampling localities with high genetic diversity (π>0.005) that are composed of more than one MOTU per species (Figs. 2, 4), i.e. demonstrating co-occurrences of deep intraspecific genetic lineages. These *Carychium* populations could be either formed in zones of secondary contact between postglacial expanding MOTUs or they constitute mixed remnant populations comprising lineages from multiple interglacial expansion events which survived the last glacial period in cryptic microrefugia [7]–[9]. The first scenario seems likely for regions along the Rhine River basin, with likely passive transportation of *Carychium* lineages along the northward-flowing river system (Fig. 4). A high relative probability score for CM_MOTU1_ and, in the case of CT, the occurrence of three highly divergent MOTUs within the region of the Sudetes could be interpreted in terms of the location of cryptic microrefugia within this region (Figs. 4B, 7A).

To sum up, our results provide consistent, independent evidence for the Alpine Region (Alps+Dinaric Alps) serving as an origin of genetic diversity (Alpine Region ‘hot-spot’ hypothesis). This condition was most likely achieved through high habitat heterogeneity and varying habitat suitability in this mountainous area [30]. During the postglacial period, accompanied by changing climatic conditions, several Alpine lineages of both *Carychium* taxa were able to expand into surrounding areas to occupy previously unsuitable regions. The recolonization of northern Europe was only achieved by a single lineage in both taxa.

### Anthropogenic impact on distribution and phylogeographical patterns

Since humanity has moved around the planet, ongoing globalization via migration, urbanization, and air and ship traffic has fostered homogenization of communities while simultaneously obliterating primal phylogeographical patterns [82]–[86]. Such leveling was also observed in the case of *Carychium* microgastropods. Originally endemic to Europe and Western Asia, populations of CM and CT have been recorded from the Azores and from North America [29], [87], [88]. Since *Carychium* microgastropods are prone to phenotypic plasticity and taxonomic identifications based on conchology alone are oftentimes inadequate for this taxonomic group [31], [89], these findings hereby only compose the molecularly confirmed sampling localities of our study (Table 1). Besides additional sightings from North America [90]–[94], potential occurrence records have been made for North Africa [95], Israel [96] and the archipelago of Madeira [97]. As part of this study, we were able to provide the first molecular evidence of transatlantic CM and CT present on the Azores and in North America.

The high similarity or identity of haplotypes found on the Azores and in North America to European MOTUs of CM and CT suggest a recent origin (Table 1, Fig. 3). A mode of passive dispersal is most likely since neither the Azores nor North America exhibit geographical affinity to European *Carychium* populations. Moreover, these microgastropods are well-known entities of European greenhouses [98], [99] and several studies exist where an anthropogenic introduction of European gastropods to North America has been suggested [82], [83], [94], [100], [101]. Such a scenario seems equally reasonable for the transatlantic populations of CM and CT. The transatlantic dispersal of CM populations can parsimoniously be ascribed to two separate events: Specimens found on the Azores, Portugal and in Ithaca, USA cluster within a single haplotype lineage (CM_MOTU4_), which otherwise is restricted to the broader peri-Alpine region in Europe (Table 1, Figs. 3A, 4A). Although assigned to CM_MOTU2_, specimens obtained from Pittsburgh, USA were genetically highly divergent from any other known European haplotype (Fig. 3A). Thus, a potential origin for this introduction could not be deduced so far. All three transatlantic localities of CT revealed haplotypes included in the most widespread and abundant European MOTU (CT_MOTU1_) (Table 1, Fig. 4B), providing no further resolution but suggesting a single event of passive dispersal from Europe to the Azores with continuation to North America. If the invasion events of the Azores and North America are not linked between as well as within both species, we detect several events of passive transportation. Finally, the patchy distribution of occurrence records of European *Carychium* species in North America (e.g. East Coast, Great Lakes region and the West Coast) potentially indicates a multitude of human-mediated passive dispersal events.

In conclusion, passive (anthropogenic) dispersal must be equally considered as a likely factor contributing to the formation of apparent phylogeographical patterns in Europe. However, we can not conclusively verify whether the *Carychium* populations on the European mainland reached and expanded into their postglacial distributions by either actively migrating or passively dispersing via wind and water [20], [102], [103], large mammals, humans or birds [83], [104]. Still, our data supports a mixture of scenarios in which *Carychium* microgastropods actively migrated and were passively dispersed by wind (on a small scale), water (e.g. along the Rhine River), birds and large mammals including humans (most probable for postglacial recolonization of Northern Europe and the basis of transatlantic populations).

## Supporting Information

Table S1 Bayes Factor (BF) tests for refugium localization reconstruction (RLR) approach. All values had an effective sample size (ESS) greater than 100. At least 10,000,000 generations were run. Tree sampling was conducted each 1,000^th^ generation. After [46], the results of log BF can be interpreted as substantial (½-1), strong (1–2) and decisive evidence (>2) for a given hypothesis.
(DOCX)

Table S2 Refugium localization reconstruction for expanding lineages of *Carychium minimum* (CM) and *Carychium tridentatum* (CT). Locality abbreviations correspond to Table 1 and Fig. 1. The relative probability score (RPS) is provided for each character (i.e. locality, pooled localities) in a given lineage. MOTU = Molecular Operational Taxonomic Unit. Significant values are underlined and marked in bold.
(DOCX)
