# Supplementary material for: Alpine Crossroads or Origin of Genetic Diversity? Comparative Phylogeography of Two Sympatric Microgastropod Species
Source: PLoS One. 2012 May 14;7(5):e37089. doi: 10.1371/journal.pone.0037089 (PMC3351404; doi:10.1371/journal.pone.0037089)
Supplement: Table S1 — Bayes Factor (BF) tests for refugium localization reconstruction (RLR) approach. All values had an effective sample size (ESS) greater than 100. At least 10,000,000 generations were run. Tree sampling was conducted each 1,000th generation. After [46], the results of log BF can be interpreted as substantial (½-1), strong (1–2) and decisive evidence (>2) for a given hypothesis. (DOCX) [file pone.0037089.s001.docx]

| parameters | taxon | | | |
| --- | --- | --- | --- | --- |
|  | CM_MOTU1_ | CM_MOTU4_ | CT_MOTU1_ | CT_MOTU5_ |
| tree likelihood_constant size_ | -985.749 | -879.100 | -931.565 | -841.893 |
| tree likelihood_exponential growth_ | -987.656 | -882.636 | -932.619 | -845.600 |
| log BF | 1.907 | 3.536 | 1.054 | 3.707 |
| final burn-in rate  (constant size model) | 2,000,000 | 1,000,000 | 3,500,000 | 2,000,000 |
