# Supplementary material for: Alpine Crossroads or Origin of Genetic Diversity? Comparative Phylogeography of Two Sympatric Microgastropod Species
Source: PLoS One. 2012 May 14;7(5):e37089. doi: 10.1371/journal.pone.0037089 (PMC3351404; doi:10.1371/journal.pone.0037089)
Supplement: Table S2 — Refugium localization reconstruction for expanding lineages of Carychium minimum (CM) and Carychium tridentatum (CT). Locality abbreviations correspond to Table 1 and Fig. 1. The relative probability score (RPS) is provided for each character (i.e. locality, pooled localities) in a given lineage. MOTU = Molecular Operational Taxonomic Unit. Significant values are underlined and marked in bold. (DOCX) [file pone.0037089.s002.docx]

| Character / Taxon | 1 | 2 | 3 | 4 | 5 | 6 | 7 | 8 | 9 | 10 | 11 | 12 | 13 | 14 | 15 | 16 | 17 | 18 | 19 | 20 | 21 | 22 | 23 | 24 |
| --- | --- | --- | --- | --- | --- | --- | --- | --- | --- | --- | --- | --- | --- | --- | --- | --- | --- | --- | --- | --- | --- | --- | --- | --- |
| CM_MOTU1_ | FR  LG  DA  BH | HU | BO  SL  RE  BY | ER | BA | WH | SZ | BR | ZU | MO | UP | PH  FV | TO  GA | EP | GH | SO  HW | WE | BT |  |  |  |  |  |  |
| RPS | 6.9 | 0.2 | **35.9** | 1.5 | 1.7 | 1.5 | 1.0 | 2.7 | 0.1 | 0.3 | 2.0 | 1.4 | **38.8** | 0.8 | 0.2 | 4.1 | 0.1 | 0.8 |  |  |  |  |  |  |
| CM_MOTU4_ | PL  PO | LG  KE  BH  ER | PF  ZU | DI | AZ | OT | WE | IT | CS | MA |  |  |  |  |  |  |  |  |  |  |  |  |  |  |
| RPS | **34.0** | 2.3 | 14.1 | 12.4 | 1.5 | 6.4 | 2.5 | 11.7 | 7.1 | 7.9 |  |  |  |  |  |  |  |  |  |  |  |  |  |  |
| CT_MOTU1_ | FR  KE  DA  ER  ES | LF  OB  BI | WU  SG  GN | OF | LM  LT  LB  LC | SM  SH | AZ | EC | HA  GM  PG  OT  BB | BA | LO  BE | WE | GI | BR | BT | LP | VA | EU  PH  FV  AS | GR | HU | VR | TS  BN | WH | PA  AU |
| RPS | 8.9 | 3.6 | 5.4 | 0.4 | **34.3** | 15.5 | 1.4 | 0.0 | 7.6 | 2.9 | 4.0 | 0.2 | 0.0 | 0.3 | 0.4 | 0.1 | 0.2 | 4.8 | 0.0 | 2.8 | 0.4 | 4.0 | 0.5 | 2.3 |
| CT_MOTU5_ | LM  AR | BO  EC  CE | RE | DA | PG | BA | AU  EP | PR | LP  LD | LA  SC | LS |  |  |  |  |  |  |  |  |  |  |  |  |  |
| RPS | 8.6 | 25.7 | 0.3 | 1.0 | 0.0 | 1.2 | 23.5 | 0.5 | 19.7 | 18.4 | 1.2 |  |  |  |  |  |  |  |  |  |  |  |  |  |
